# Mitochondrial-mediated apoptosis as a therapeutic target for FNC (2′-deoxy-2′-b-fluoro-4′-azidocytidine)-induced inhibition of Dalton’s lymphoma growth and proliferation

**DOI:** 10.1007/s12672-023-00829-6

**Published:** 2024-01-22

**Authors:** Naveen Kumar, Sanjeev Kumar, Alok Shukla, Sanjay Kumar, Rishi Kant Singh, Ilya Ulasov, Sandeep Kumar, Anand Kumar Patel, Lokesh Yadav, Ruchi Tiwari, Rachana, Shivashish Priyadarshi Mohanta, Kaushalendra, Vikram Delu, Arbind Acharya

**Affiliations:** 1 Department of Zoology, Institute of Science, Banaras Hindu University, Varanasi, Uttar Pradesh 221005 India; 2 Department of Zoology, Lucknow University, Lucknow, Uttar Pradesh 226007 India; 3 Group of Experimental Biotherapy and Diagnostic, Department of Advanced Materials, Institute for Regenerative Medicine, Sechenov First Moscow State Medical University, Moscow, 119991 Russia; 4 World-Class Research Center, Digital Biodesign and Personalized Healthcare, Sechenov First Moscow State Medical University, Moscow, 119991 Russia; 5 Department of Zoology, Pachhunga University College Campus, Mizoram University, Aizawl, Mizoram 796001 India; 6 Haryana State Biodiversity Board, Panchkula, Haryana 134109 India

**Keywords:** Haematology, Histology, Viability, Fluorinated nucleoside analogues, Apoptosis

## Abstract

**Purpose:**

T-cell lymphomas, refer to a diverse set of lymphomas that originate from T-cells, a type of white blood cell, with limited treatment options. This investigation aimed to assess the efficacy and mechanism of a novel fluorinated nucleoside analogue (FNA), 2′-deoxy-2′-β-fluoro-4′-azidocytidine (FNC), against T-cell lymphoma using Dalton’s lymphoma (DL)-bearing mice as a model.

**Methods:**

Balb/c mice transplanted with the DL tumor model received FNC treatment to study therapeutic efficacy against T-cell lymphoma. Behavioral monitoring, physiological measurements, and various analyses were conducted to evaluate treatment effects for mechanistic investigations.

**Results:**

The results of study indicated that FNC prevented DL-altered behavior parameters, weight gain and alteration in organ structure, hematological parameters, and liver enzyme levels. Moreover, FNC treatment restored organ structures, attenuated angiogenesis, reduced DL cell viability and proliferation through apoptosis. The mechanism investigation revealed FNC diminished MMP levels, induced apoptosis through ROS induction, and activated mitochondrial-mediated pathways leading to increase in mean survival time of DL mice. These findings suggest that FNC has potential therapeutic effects in mitigating DL-induced adverse effects.

**Conclusion:**

FNC represents an efficient and targeted treatment strategy against T-cell lymphoma. FNC’s proficient ability to induce apoptosis through ROS generation and MMP reduction makes it a promising candidate for developing newer and more effective anticancer therapies. Continued research could unveil FNC’s potential role in designing a better therapeutic approach against NHL.

## Introduction

Non-Hodgkin lymphomas (NHLs) are a global health concern, with an estimated 95,000 new cases in the US in 2022 [41]. NHL disrupt lymphocyte function and spreads through the lymphatic and circulatory systems. T-cell lymphoma is a distinct NHL subtype originating from abnormal T lymphocytes. T-cell lymphoma pose significant challenges, affecting multiple organs and displaying aggressive behavior. Treatment options include chemotherapy, immunotherapy, targeted therapy, and radiation therapy [21, 38]. However, these therapies have limitations, necessitating further research for improved strategies [45]. Notably, fluorine-containing drugs, known as FNAs, play a critical role in NHL treatment [27]. FNAs, such as antivirals and anticancer therapeutics, induce cell cycle arrest, promote apoptosis, and inhibit DNA synthesis [15]. They are particularly effective in relapsed and refractory cases [48].

**Fig. 1:**
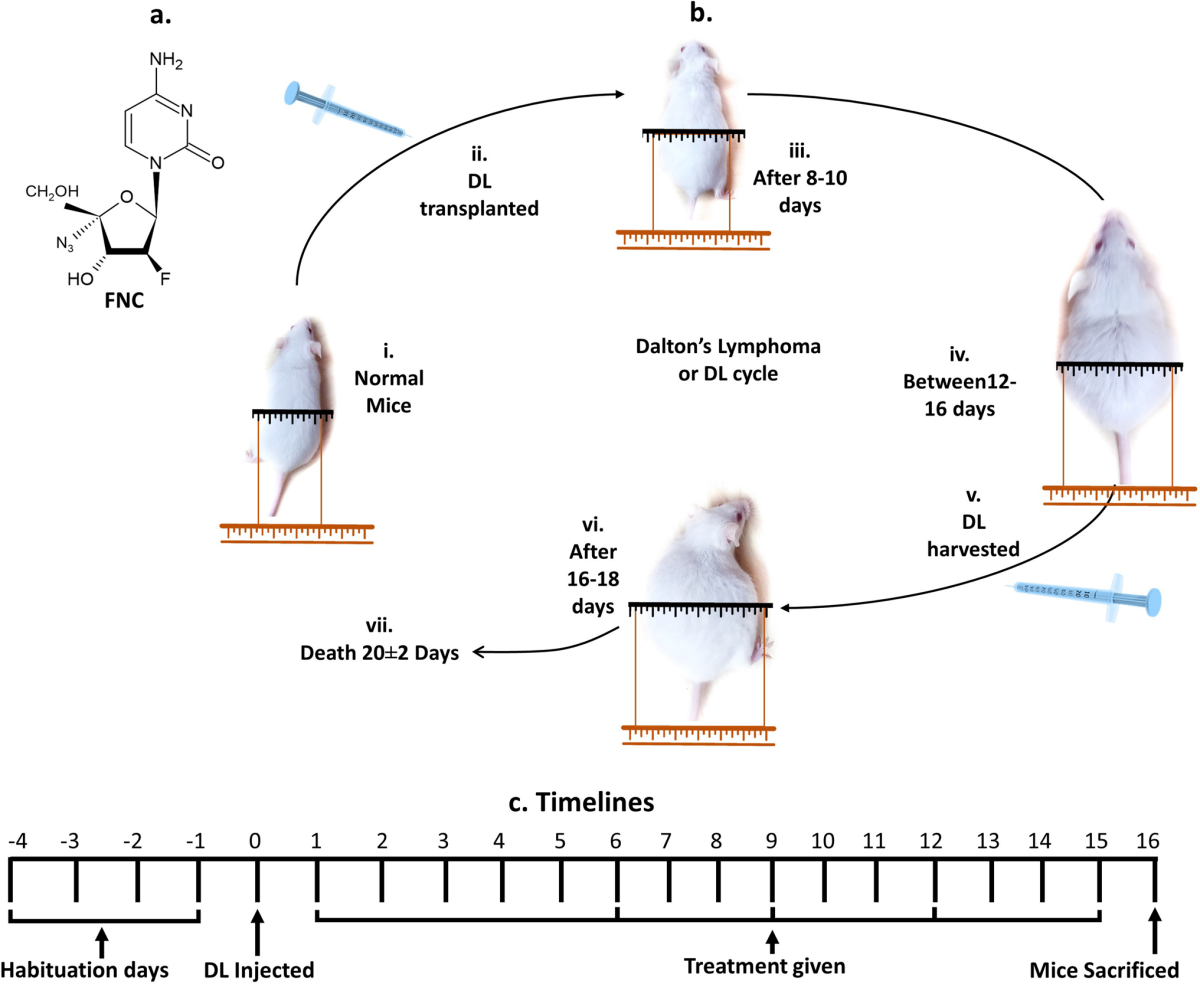
Structure of 2′-deoxy-2′-β-fluoro-4′-azidocytidine (FNC), Dalton’s lymphoma (DL) cycle, and the experimental timeline for treatment. **a** Chemical structure of FNC, the drug employed for treating vehicle-treated DL-bearing group. **b** DL cycle sustained through serial transplantation of DL cells in healthy mice. (i, ii) DL growth was initiated using 1 × 10^6^ DL cells. (iii–v) A period of minimal growth was observed within 8–10 days, followed by a surge in growth between 12–16 days, which was considered optimal for harvesting. (vi) The peak DL growth was attained at 16–18 days; however, harvesting at this stage was not recommended. (vii) Mice experienced mortality after 20 ± 2 days. The DL belly size growth depicted on the hypothetical scale is solely for illustrative purposes. **c** Timeline of treatment or experimental protocol: Mice were habituated for 4 days, followed by transplantation with DL cells after overnight fasting. Subsequently, the mice were treated with vehicle, the standard drug gemcitabine at 10 mg/mL (G), FNC at 5 mg/mL (F5), or FNC at 10 mg/mL (F10) every third day for a total of 15 days. On day 16, the mice were sacrificed for further analysis

In this regard, FNC is a recently synthesized FNA, clinically approved with the brand name Azvudine, which has shown remarkable therapeutic efficacy against both cancer and viral diseases, making it a promising candidate for clinical applications. FNC’s structure is analogous to that of normal cytidine with a unique 4′-Azido (N3) and 2′-fluorinated deoxynucleotide composition (Fig. 1a). The fluorine atom located at the 2′-position of FNC confers its exceptional property of competing with natural nucleotides, which is responsible for its inhibitory effect on cell growth and proliferation [24, 53]. FNC’s ability to disrupt DNA synthesis by integrating into DNA and RNA has been demonstrated in several studies involving various cancers such as acute myeloid leukemia [47], non-small-cell lung cancer cells, lung adenocarcinoma [13], sarcoma, gastric carcinoma, and hepatocarcinoma [47]. Furthermore, FNC has been approved for treating individuals with high HIV viral loads, further highlighting its versatility and potential for a wide range of clinical applications [9].

Previous studies have reported the efficacy of FNC in inhibiting the proliferation and metastasis of various tumor cell lines, including NHLs [47, 54, 55]. FNC has been shown to induce cell cycle arrest and apoptosis in B-cell origin NHL cell lines [47], as well as inhibit the growth of NHL cell lines, Raji and JeKo-1, by activating apoptotic factors and targeting proteins related to cell invasion, migration, and adhesion [54]. In vivo studies have also demonstrated the efficacy of FNC in significantly reducing tumor growth in mice xenograft models of sarcoma, hepatocarcinoma, and gastric carcinoma, with efficacy similar to that of clinical drugs such as capecitabine [47], cisplatin [13], and cytarabine [55], respectively. However, the effectiveness of FNC against T-cell lymphoma remains unknown. Therefore, we evaluated the therapeutic potential of FNC in treating T-cell lymphoma using a murine model of DL. DL is a useful model for testing new drugs in the drug discovery and development process [18].

## Materials and methods

### Materials

FNC and Gemcitabine were obtained from Granlen Inc., China, and Sigma-Aldrich, USA, respectively. Thermo-Fisher Scientific, USA served as the source for various reagents, including 4′,6-diamidino-2-phenylindole (DAPI), Acridine orange (AO), Ethidium bromide (EtBr), 2′-7′-Dichlorodihydrofluorescein diacetate (DCFDA), Rhodamine (Rh-123), trypan blue, antibodies, and the caspase-3 and -9 kit. The apoptosis kit Annexin-V FITC and Propidium iodide (PI) were purchased from Invitrogen, USA, while all other chemicals were procured from the Hi-media laboratory located in Mumbai, India. The quality and purity of these reagents were ensured through proper checking before order or use, and all experiments were performed using high-quality, standardized materials and procedures to ensure the accuracy and reproducibility of the results.

#### Tumor model

Pathogen-free Balb/c (H2d) mice were selected as animal models due to their widely accepted use in preclinical research. Mice aged 8–12 weeks of either sex and weighing 30 ± 2 g were used to minimize variability in the study. The DL tumor model was chosen as it closely mimics the characteristics of T-cell lymphoma in humans, making it an ideal model for studying novel treatments [18]. To induce DL, mice were transplanted intraperitoneally (*i.p.*) with 1 × 10^6^ DL cells in 1 mL chilled Phosphate-Buffered Saline (PBS), following the protocol described elsewhere (Fig. 1b) [40]. This method ensures a high rate of DL proliferation and growth, providing a reliable model for the assessment of treatment efficacy. The use of *i.p.* transplantation also allows for easy monitoring of tumor growth and metastasis through visible growth in mice belly and overall weight. Overall, the selection of the appropriate animal model is crucial in preclinical research, and the use of the DL tumor model in this study provides a strong foundation for assessing the effectiveness of FNC as a potential treatment for T-cell lymphoma. This study was approved and conducted according to the guidelines of the Institutional Animal Ethics Committee of Banaras Hindu University (BHU), Varanasi, India.

#### Treatment protocol of animals

The study implemented a randomized animal model consisting of five groups, each comprising six pathogen-free Balb/c (H2d) mice (n = 6) (Table 1). The experiment followed a rigorous timeline (Fig. 1c) and involved transplanting the mice with DL on day 0, after overnight fasting, and drug treatment *i.p.* every third day up to day 15. The highest dose of 10 mg/kg was selected based on the results of a recent acute toxicity study, which demonstrated that FNC up to 10 mg/kg body weight was non-toxic to the mice (Article in Press). The drugs FNC and Gemcitabine were dissolved in Dimethyl sulfoxide (DMSO) and diluted in PBS. On day 16, the mice were humanely euthanized under light anesthesia and through painless cervical dislocation.

**Table 1:** Animals groupings (n = 6)

Groups	Group name	Dose given (total 1 mL *i.p.*)
Group I	Normal mice (N)	PBS only
Group II	Negative control (V)	Vehicle-treated
Group III	Positive control (G)	10 mg/kg body weight gemcitabine
Group IV	Low dose (F5)	5 mg/kg body weight FNC
Group V	High dose (F10)	10 mg/kg body weight FNC

### Behavioral parameters

#### Behavioral parameters

The mice groups were closely monitored for any changes in behavioral parameters for 2 h a day, ensuring any potential adverse effects were promptly identified. The groups were provided with 50 mL of water and 25 g of feed daily, and group consumption was recorded after 24 h.

#### Body weight, belly size, organ weight, and coefficient measurement

In order to assess the treatment effects of FNC on DL mice, various physiological parameters were measured. The body weight and belly size of the different mice groups were recorded every third day to monitor any changes due to the treatment. On the day of sacrifice, the mice’s organs were removed, washed with PBS, and dried over tissue paper. The weight of each organ was recorded, and the organ coefficient was calculated using the given equation. These measurements allowed for the assessment of any potential toxicity or adverse effects of FNC on the mice.

$$Organ \;cofficient= \frac{Weight \;of\; organ \;(\mathrm{g})}{Total\; body\; weight\; of\; mice \;(\mathrm{g}) }\times 100$$

### Hematological and biochemical parameters examination

Hematological analyses were performed by collecting blood samples from the mice’s tail veins in both EDTA and non-EDTA-coated vials. These blood samples were then provided to a commercial laboratory, Parul Pathology, Varanasi, for the analysis of blood parameters, cytokines, Interleukin-6 (IL-6) and Tumor Necrosis Factor-alpha (TNF-α), and liver and renal function tests (LFT & RFT). The results of these analyses provided valuable insights into the efficacy and safety of the drug FNC.

### Histopathological examination

The organs collected from the mice were meticulously evaluated via histopathological examination to decipher any underlying structural alterations. A 10% formalin solution was used to fix the isolated organs, which were then dispatched to Parul Pathology for slide preparation and staining with hematoxylin and eosin (H&E). The resulting slides were examined under bright field mode on a high-resolution fluorescence microscope (Olympus BX63) to ascertain any metastatic foci. The angiogenic potential was evaluated by capturing images of the abdominal wall and tumor and manually quantifying the number of blood vessels. This meticulous analysis was performed to gain insights into the structural and angiogenic alterations that may have arisen due to drug treatments.

### Supplementary tests

On day 16, prior to the sacrifice of the mice, the peritoneal fluid containing DL cells was obtained, washed thrice, and subjected to further experimentation. Minute fragments of the mice’s liver and spleen were finely minced to form a single-cell suspension using well-established protocols  [28, 44], and the cell viability was assessed through the Dilution-cum-trypan (DCT) assay. This assay is our recently developed technique, as an alternative to conventional trypan assay, and provides an accurate and sensitive measure of cell viability. The results of this assay provide crucial insight into the overall effectiveness of the treatment and the extent of the impact on the cells.

#### DCT assay for cell viability and proliferation

To assess cellular proliferation, cells extracted from the liver, spleen, and peritoneum of mice were subjected to a DCT assay. After staining the cells with a DCT standard trypan concentration (0.4%) for 3 min, the cells were washed, and viability was determined by calculating the number of live cells in relation to the total number of cells. Additionally, the total number of cells obtained from the peritoneal fluid on day 16 was also stained with DCT, and the resulting live cell counts were used to assess cellular proliferation.

$$Cell\; viability= \frac{Number\; of\; live\; cells}{Total\; number\; of\; cells }\times 100$$

#### Fluorescent tests

In order to perform fluorescent tests, extracted DL cells were washed and mixed directly with dyes in a 30 µL solution. DAPI, a fluorescent DNA stain (1 mg/mL), was used after fixing the cells with 4% paraformaldehyde for 5 min. Other staining agents, such as DCFDA, Rh-123, and AO/EtBr at 20 µg/mL, were used singly or in combination depending on the test and were applied for 5–10 min. Images were captured using a fluorescence microscope at 20× magnification. For Annexin/PI staining, cells were washed with Annexin-binding buffer and stained according to the manufacturer’s instructions. Moreover, Annexin/PI, ROS, and MMP were analyzed using flow cytometry (CytoFLEX LX). Methylene blue staining was performed by fixing cells in 50% ethanol for 10 min and staining with a 0.2% (v/v) methylene blue solution prepared in ethanol. A total of 300 cells were counted for all indexes. These tests were used to assess the viability, proliferation, and functional characteristics of DL cells in response to treatment.

#### Quantitative image analysis via DAPI staining

The Box-Counting Fractal Dimension (BOX-FD) analysis, as described by Chalut et al. [4], was used in this study to quantify apoptotic and viable cells using DAPI-stained microscopic images [4]. A fluorescence microscope with a 40× objective lens was utilized to capture images of 40 live and dead cells, and their BOX-FD was analyzed using the built-in function of Image J software. The BOX-FD, a measure of the complexity and shape of the image, was calculated based on the number of squares required to cover the image, with higher values indicating a more complex structure and lower values indicating a less complex structure. This analysis provides valuable information about the apoptotic and viable cells, as changes in the shape and complexity of the cell images can be quantified and assess the efficacy of potential therapeutic interventions.

#### Scanning electron microscopy (SEM)

The study employed a rigorous sample preparation protocol for SEM, where the cells were fixed in glutaraldehyde prepared in 0.1 M PBS for 30 min, followed by post-fixation with OsO4 overnight. Subsequently, the sample was dehydrated using a series of ethanol and coated with gold-palladium (Quantum technology-SC7620) for imaging under the SEM (Zeiss-EVO LS-10) at 25 kV at LV at the Department of Zoology, Banaras Hindu University, India. This meticulous sample preparation procedure allowed for the detailed examination and characterization of the cellular structure and morphology, thereby providing valuable insights into the apoptotic effects of the drug.

#### Western blots analysis

Protein extraction from washed cells was carried out using the Radio-Immunoprecipitation Assay buffer (RIPA) buffer, followed by the separation of 30 µg protein per sample using 12% SDS-PAGE. The protein was transferred onto a Polyvinylidene Fluoride membrane (PVDF) membrane and blocked with 5% skimmed milk. Subsequently, the membrane was incubated overnight with primary antibodies and washed with Tris-Buffered Saline with Tween (TBST). A Horseradish Peroxidase (HRP)-conjugated secondary antibody was added and incubated at room temperature for 1 h. The protein bands were visualized using enhanced chemiluminescence (ECL) and their relative intensity was quantified using Image J software. Furthermore, the activity of caspase-9 and -3 was measured according to the manufacturer’s instructions. This comprehensive approach enabled us to accurately evaluate the level of protein expression and activation of caspase-9 and -3, key players in apoptosis. Such assessment of protein activity provides crucial insights into the complex mechanisms governing cell death.

### Survival study

To assess the efficacy of FNC as a treatment for T-cell lymphoma, the mice were observed for 60 days, and survival data were recorded and plotted using the Kaplan–Meier survival curve. The percentage increase in life span was calculated using a formula that compares the number of days treated mice (T) and DL control mice or vehicles (V) survived. This calculation allowed us to accurately measure the effectiveness of FNC treatment in extending the life span of mice with T-cell lymphoma.

$${\%} \;\mathrm{Increase}\; \mathrm{in}\; \mathrm{life}\; \mathrm{span} = \frac{T-V}{V }\times 100$$

### Statistical analysis

Data analysis was conducted using the GraphPad Prism 5.01 software (GraphPad Software, CA). The results are presented as mean ± standard deviation (SD) for each group unless otherwise stated. The statistical significance of the differences between the two groups was assessed by an unpaired t-test, while the significance of the differences among more than two groups was assessed by a one-way or two-way Analysis of variance (ANOVA) followed by Tukey or Bonferroni post-test, respectively. We use the log-rank test to determine the significance of the Kaplan–Meier curves. The significance level was set at *p < 0.05, and * and ^○^ denote a significant difference between Normal mice vs. F, F5, and F10, and Vehicle group vs. G, F5, and F10, respectively. These statistical analyses ensure the robustness and reliability of the results, facilitating a clear interpretation of the experimental outcomes.

## Results

### FNC reverted DL-altered behavior parameters

DL growth can induce severe behavioral alterations in mice, which are among the primary parameters affected [7, 11]. These changes in the behavior of vehicle-treated DL-bearing group (Group-II) include reduced food intake and increased water consumption, among others (Table 2). However, interestingly, FNC treatments group F5 and F10 (Groups-IV, and V, respectively) was observed to normalize these behavioral changes induced compared to the vehicle group (p < 0.05), thus suggesting its potential to mitigate the adverse effects caused by cancer growth.

**Table 2:** Impact of FNC on the DL altered behavior of mice

Parameters	V	G	F5	F10
Feed intake	−−	Ns	−	−
Water intake	++	−	+	Ns
Movement	−−−	−	−−	−
Eye bulging	++	+	++	+
Eye color	++	Ns	+	Ns
Body weight	++++	+	++	+
Belly size	++++	+	++	+
Cuddling	+	Ns	+	Ns
Sleeping	+++	+	++	+
Appearance of fur	−−	Ns	−	Ns
Cleanliness of coat and body	−−	Ns	−	−
Urination	++	+	+	+
Fecation	++	+	+	++

The symbols (+), (−) and Ns represent the increase, decrease or not seen in the experimental groups, respectively compared to the control group

#### FNC prevented the DL to increase the mice’s body weight and belly size

**Fig. 2:**
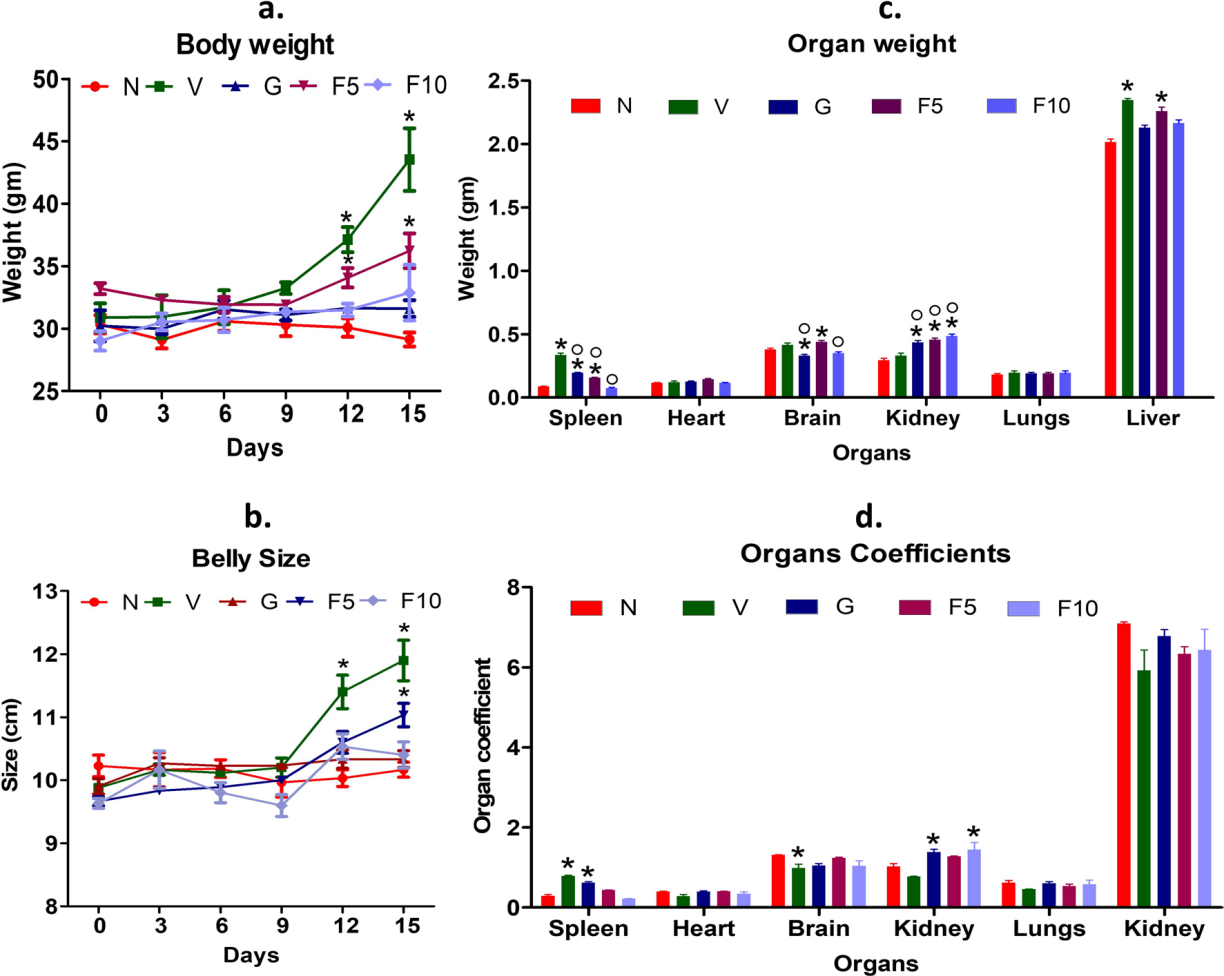
Analysis of multiple parameters in mice, including body weight, belly size, organ weight, and organ coefficient. **a** Changes in the body weight of mice were measured every third day (0, 3, 6, 9, 12, and 15). **b** Measurement of belly size in mice at the same time points. **c** Extraction of organs from mice and recording of their weights for the determination of the organ coefficient. **d** Illustration of the organ coefficient, calculated by measuring the ratio of organ weight to body weight. Measurements of mouse body weight and belly size were recorded on days 0, 3, 6, 9, 12, and 15. Organ weights were also measured, and the organ coefficient was determined by relating the organ weight to the body weight. n = 6. Symbolic notation ^○^ and * were used to indicate significant differences (p < 0.05), between normal mice (N) vs. V, G, F5, F10, and V vs. G, F5, F10, respectively

In earlier DL studies, it was demonstrated that DL cells proliferate in the peritoneum, leading to a significant increase in both body weight and belly size of mice [5]. The study revealed that vehicle-treated DL-bearing group started to experience rapid weight gain due to the rapid proliferation of DL cells after 10 days and attained their peak weight and belly size 1 day before they sacrificed (day 15) compared to normal mice (p < 0.05) (Fig. 2a, b). The FNC treatment had a significant dose-dependent effect in preventing the DL ability to increase body weight and belly size of mice compared to the vehicle group. However, F10 group, itself, don’t have any impact on the mice weight as found in our acute toxicity study. In addition, the F5 and F10-group mice had body weights and belly sizes that were comparable to G-group mice, which were significantly lower than that of the vehicle group (p < 0.05).

#### FNC prevented the DL to increase spleen and liver weights

DL growth and metastasis have been found to have a significant impact on the spleen and liver weight in mice, as reported in studies [35, 44]. The mean spleen and liver weight in vehicle-treated DL-bearing group was significantly higher compared to normal mice. However, the administration of FNC was observed to have a beneficial effect in preventing the DL-induced weight gain in spleen and liver in a dose-dependent manner compared to the vehicle group (p 0.05) (Fig. 2c). The F10-group found to be particularly effective in preventing the liver weight gain, which was similar to the G-group (Group-III) compared to the vehicle group (p 0.05). In contrast, all three treatment groups (G, F5, F10) showed a significant increase in kidney weights, although only the F10 and G-group exhibited a significant increase in kidney coefficient analysis compared to the vehicle group (Fig. 2d).

### FNC prevents the DL-caused alteration in haematological parameters

The immune system plays a significant role in cancer pathogenesis, and therefore, the haematological parameters are important for monitoring cancer development and treatment efficacy [6, 14]. The aim was to investigate the effect of DL and how FNC treatment can mitigate DL-induced alteration in haematological parameters in mice (Table 3). The study results showed that DL growth induced a significant increase in WBC, platelets, neutrophils, and MCV in vehicle-treated DL-bearing group, while FNC treatment prevents these DL-induced changes compared to the vehicle group (p < 0.05). The G-group had intermediate level of haematological parameters lies between the vehicle group and normal mice (p < 0.05). Additionally, DL growth decreased the levels of Hb, lymphocytes, and MCHC in the vehicle group, while FNC treatment prevented their decrease compared to the vehicle group (p < 0.05). The G-group prevents the tumor-induced lymphocytes and Hb increase but did not affect MCHC compared to the vehicle group (p < 0.05). Previous studies suggest that DL tumor progression can lead to changes in haematological parameters, including increased WBC and neutrophil counts and decreased lymphocyte counts, due to the suppression of normal T-cell function and the activation of innate immune responses by tumor-associated antigens [1, 8].

**Table 3:** Hematological parameters of different groups

Hematological parameters					
Parameter	N	V	G	F5	F10
RBC (×10^6^/µL)	5.72 ± 0.224	6.21 ± 0.732	6.40 ± 1.122	5.50 ± 0.14	6.91 ± 0.632
WBC (×10^3^/µL)	6.60 ± 0.266	25.60 ± 1.76*	13.70 ± 1.121*^○^	17.60 ± 3.83*^○^	14.10 ± 1.23*^○^
Platelets (×10^5^/µL)	1.32 ± 0.213	5.11 ± 0.226*	**2.41 ± 0.362**^○^	**1.82 ± 1.24**^○^	**1.57 ± 0.783**^○^
Lymphocyte %	78 ± 4.2	40 ± 2.216*	64 ± 5.32*^○^	**66 ± 8.83**^○^	53 ± 5.23*^○^
Neutrophil %	14 ± 1.65	52 ± 4.226*	**18 ± 4.24**^○^	25 ± 4.17*^○^	28 ± 2.89*^○^
Monocytes %	8 ± 1.123	8 ± 0.64	8 ± 0.73	9 ± 0.4	9 ± 0.5
Hb (gm/dL)	13 ± 1.12	8.1 ± 1.125*	11.9 ± 1.12	**12.8 ± 1.73**^○^	**12.72 ± 1.74**^○^
PCV %	28.4 ± 2.14	34.6 ± 1.18	31.6 ± 3.284	23.5 ± 5.73	30.4 ± 3.22
MCH (pg)	19.3 ± 1.12	17.8 ± 4.3	19.1 ± 1.29	17.8 ± 2.01	15.7 ± 2.17
MCV (fl.)	42.2 ± 3.93	55.7 ± 4.128*	45.7 ± 3.12	**42.7 ± 3.26**^○^	**41.4 ± 4.22**^○^
MCHC %	45.7 ± 2.26	32.0 ± 2.150*	34.3 ± 2.56*	30.5 ± 2.27*	**41.7 ± 2.11**^○^
RDW	17.3 ± 1.12	15.7 ± 1.43	16.6 ± 1.62	15.6 ± 2.16	15.5 ± 0.32

Data represent the mean ± SD of the group. Femtoliter (fl.), red blood cells (RBC), White blood cells (WBC), Hemoglobin (Hb), Packed cell volume (PCV), Mean corpuscular hemoglobin (MCH), mean corpuscular volume (MCV), mean corpuscular hemoglobin concentration (MCHC), Red cell distribution width (RDW). n = 6. Symbolic notation ^○^ and * were used to indicate significant differences (p < 0.05) (shown in bold), between normal mice (N) vs. V, G, F5, F10, and V vs. G, F5, F10, respectively

#### FNC cutbacks the level of tumor-induced liver enzymes

DL growth can impact the biochemical parameters of the liver or kidney to achieve maximum growth [30, 34]. Consequently, the objective here was to investigate if FNC could prevent these DL-induced changes in liver enzymes. Serum glutamic-oxaloacetic transaminase (SGOT or AST), Serum Glutamic Pyruvic Transaminase (SGPT), and bilirubin (total) levels were elevated in vehicle-treated DL-bearing group compared to normal mice (Table 4), indicating increase caused by DL growth. The increase of bilirubin (total) turned the normal mice negative Van den Bergh reaction to positive in the vehicle group, suggesting the possibility of jaundice [49]. The F10-group was assessed to determine the FNC counteracting effects to the alteration caused by DL in the liver growth parameters. Results indicated that FNC was effective in preventing SGOT and SGPT levels and decreasing bilirubin (total) levels toward normal, thereby turning the Van den Bergh reaction negative compared to the vehicle group (p < 0.05). Increased bilirubin levels in DL mice could indicate liver cirrhosis, but this was not observed in any group using anti-mitochondrial antibody tests. The RFT parameters were less significantly altered by DL growth compared to LFT parameters. However, a noTable 122% increase in cholesterol levels was observed in the vehicle group compared to normal mice, which was mitigated to 88% by F10 (Table 5) (p < 0.05). Mounting evidence suggests that high cholesterol levels may pose a dual threat to patients with both cancer and cardiovascular disease and this finding might be particularly important to test FNC in these patients.

**Table 4:** Liver function tests (LFT) of different groups

LFT					
Parameter	N	V	G	F5	F10
Serum alkaline phosphate (ALP) (IU/L)	157 ± 8.12	178 ± 25.53	158 ± 7.24	188 ± 10.12*	168 ± 9.25
Serum total protein (gm/dL)	6.8 ± 0.62	6.9 ± 1.26	7.8 ± 1.14	7.9 ± 0.5 (8.4)	6.4 ± 1.22
Serum albumin (gm/dL)	4.8 ± 0.32	3.8 ± 1.12	3.9 ± 0.42	4.6 ± 0.322	3.9 ± 1.47
Serum globulin (gm/dL)	2.0 ± 0.78	3.1 ± 0.25	3.7 ± 0.82	3.3 ± 0.36	2.5 ± 1.25
SGOT (AST) (IU/L)	97 ± 9.83	128.87 ± 12.34*	59.16 ± 12.22*^○^	**111.69 ± 17.2**	**93.26 ± 2.6**^○^
SGPT (ALT) (IU/L)	41 ± 5.24	137.45 ± 8.34*	**28.45 ± 8.72**^○^	56.45 ± 4.25*^○^	31 ± 4.38*^○^
Bilirubin total (mg/dL)	0.8 ± 0.36	4.26 ± 0.652*	**0.9 ± 0.12**^○^	2.89 ± 0.042*^○^	**0.3 ± 0.2**^○^
Bilirubin direct (mg/dL)	0.2 ± 0.08	0.6 ± 0.02	0.3 ± 0.022	0.92 ± 0.022	0.1 ± 0.06
Bilirubin indirect (mg/dL)	0.6	–	–	–	0.2
Van den Bergh reaction	−ve	+ve	−ve	+ve	−ve
Anti-mitochondrial antibody (M2) U/mL	No	No	No	No	No

Data represent the mean ± SD of the group. Serum glutamic-oxaloacetic transaminase (SGOT), Serum Glutamic Pyruvic Transaminase (SGPT). n = 6. Symbolic notation ^○^ and * were used to indicate significant differences (p < 0.05)  (shown in bold), between N vs. V, G, F5, F10, and V vs. G, F5, F10, respectively

**Table 5:** Renal function tests (RFT) of different groups

RFT					
Parameter	N	V	G	F5	F10
Urea (mg/dL)	36.14 ± 2.72	40.1 ± 4.12	42.3 ± 1.84	37.7 ± 1.63	34.10 ± 3.72
Blood urea nitrogen (BUN) (mg/dL)	19.26 ± 1.253	18.74 ± 2.94	21.14 ± 3.12	17.62 ± 7.52	15.93 ± 6.23
Creatinine (mg/dL)	0.31 ± 0.062	0.20 ± 0.012	0.36 ± 0.02	0.20 ± 0.012	0.21 ± 0.031
Sodium (mEq/L)	131.4 ± 6.22	138.5 ± 4.21	146.7 ± 11.66	149.0 ± 9.172	145.0 ± 9.123
Potassium (mEq/L)	6.8 ± 0.37	6.9 ± 0.21	6.9 ± 1.15	6.7 ± 1.62	7.9 ± 0.721
Chloride (mEq/L)	82.3 ± 7.21	108.0 ± 13.21	104.2 ± 7.12	106.6 ± 5.22	104.7 ± 6.132
Calcium (mg/dL)	9.22 ± 1.12	10.8 ± 0.182	10.3 ± 1.83	10.0 ± 1.62	10.2 ± 0.53
Phosphorus (mg/dL)	5.7 ± 0.15	8.6 ± 0.21	6.1 ± 0.72	5.9 ± 1.62	6.8 ± 1.12
Uric acid (mg/dL)	5.9 ± 0.17	2.4 ± 0.27	5.3 ± 0.92	2.9 ± 1.72	5.5 ± 0.23
Cholesterol (mg/dL)	99 ± 6.12	121.0 ± 3.20*	106 ± 4.12	**98.0 ± 4.12**^○^	**87 ± 7.15**^○^

n = 6. Symbolic notation ^○^ and * were used to indicate significant differences (p < 0.05)  (shown in bold), between N vs. V, G, F5, F10, and V vs. G, F5, F10, respectively

### FNC restores tumor-altered liver, spleen, and kidney structures

**Fig. 3:**
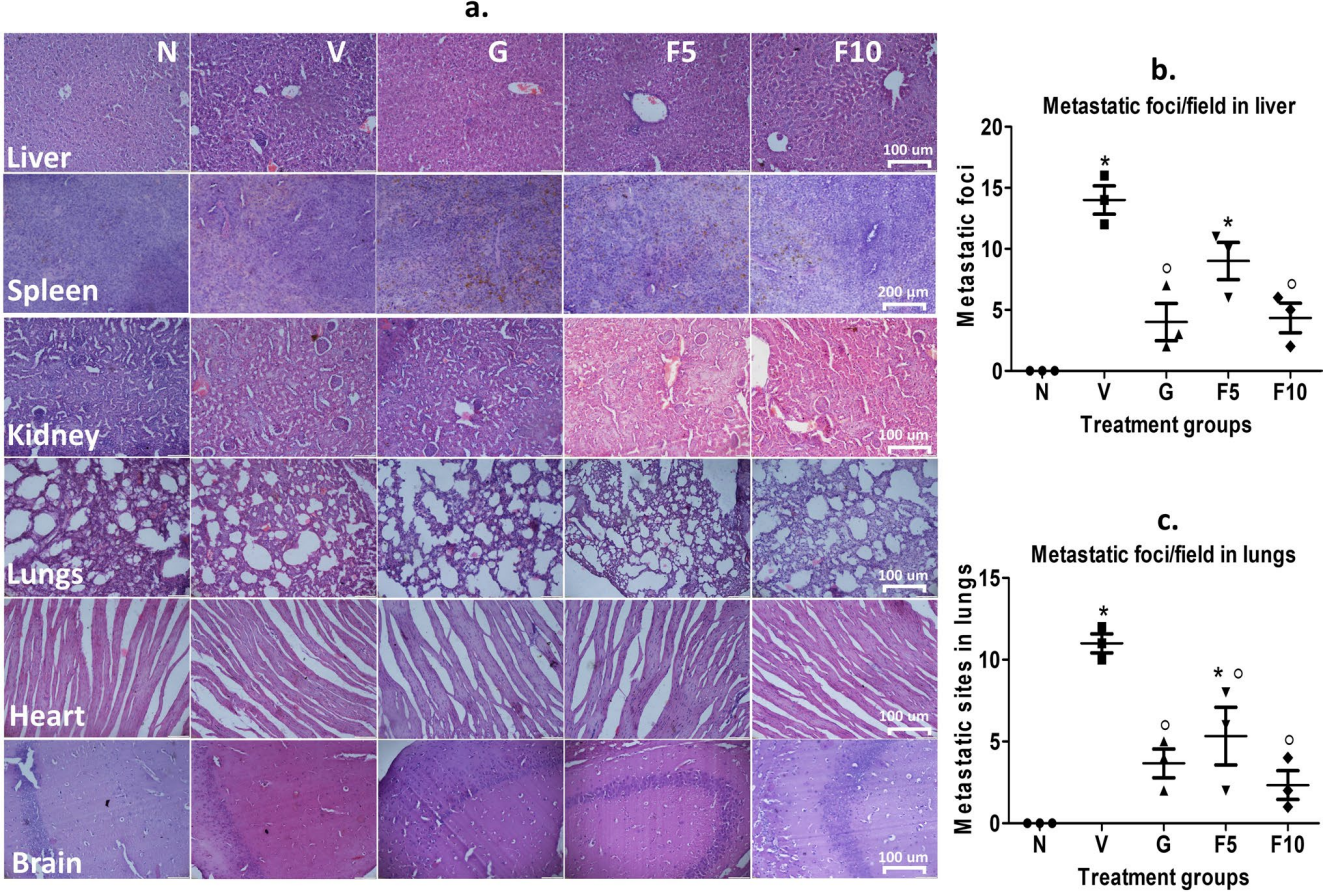
Histological examination of mice organs using H&E staining and quantification of metastasis foci. **a** H&E staining of different organs to assess the impact of DL and treatment. Representative images of the stained organs are shown in the panel. **b**, **c** Calculation of metastasis foci in the liver and lungs by carefully examining tissue structures. The scale bar used for H&E staining was 100 μm, except for the spleen, which had a scale bar of 200 μm. Symbolic notation ^○^ and * were utilized to indicate significant differences (p < 0.05) between N vs. V, G, F5, F10, and V vs. G, F5, F10, respectively

DL metastasis exerts a heavy toll on crucial organs, including the liver, spleen, and kidney, as a result of the formation of metastasis foci [12, 51]. The liver of vehicle-treated DL-bearing group displays substantial metastasis loci, along with a slight hepatocyte enlargement and accumulation of lymphocytes in portal triads. Dilated sinusoidal spaces containing lymphocytes are also present (Fig. 3a). Similarly, in vehicle group the spleen exhibits pathological changes, including distorted lymphoid architecture and the presence of granular leukocytes and giant macrophages. Moreover, the kidneys exhibited dilated renal tubules and atrophied glomeruli. In contrast, the F10-group effectively combats the organ damage induced by DL, displaying fewer or no signs of pathology and tumor sites liver and lungs (Fig. 3b, c). Earlier, the analysis of organs had indicated that FNC is more effective in safeguarding the organs (Sect. 3.1.2), and the F10 group exhibits similar tumor sites to the G-group in liver and lungs. Notably, the DL mice exhibit significant pathology in other organs, such as the lungs, heart, and brain, whereas the F10-group displays normal histology (Fig. 3a).

#### FNC reduces the pathological state of DL mice

Pathological examination revealed that DL induces discoloration in certain organs with less distinct cavities and orifices. Vehicle-treated DL-bearing group also exhibited clotting on skin peritoneal walls. In contrast, FNC-treated mice displayed fewer pathological signs in their external/internal surface, cavities, or orifices compared to the vehicle group. The F10-group did not exhibit any abnormal growth, clot, or discoloration in the body or isolated organs (data not shown).

#### FNC attenuates angiogenesis in tumor-host

**Fig. 4:**
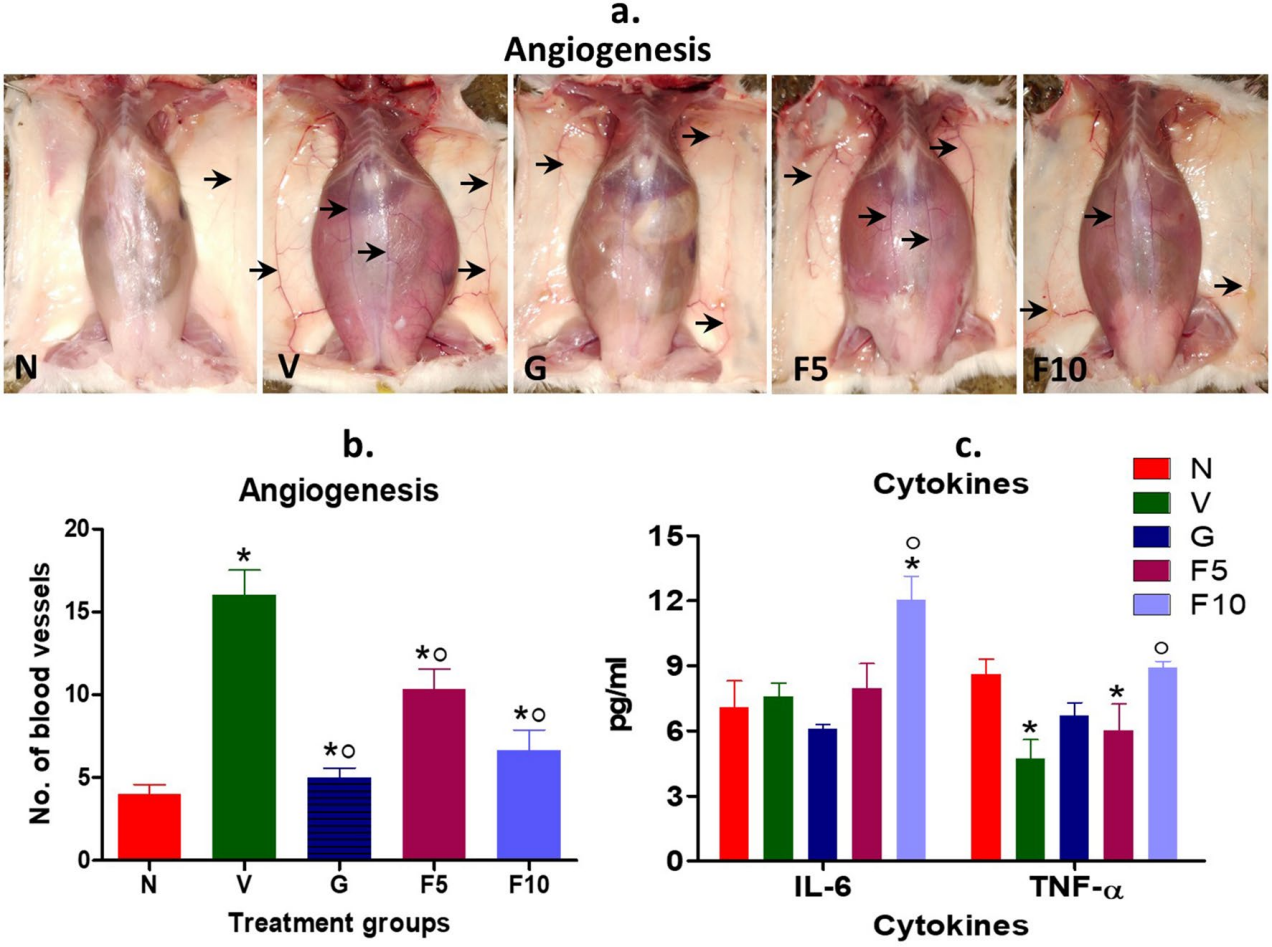
Analysis of angiogenesis and cytokines. **a**, **b** Manual scanning was employed to quantify angiogenesis in mice, with black arrows indicating blood vessels. **c** Measurement of pro-inflammatory cytokines IL-6 and TNF-α using the Beckman Coulter platform. Symbolic notation ^○^ and * were used to indicate significant differences (p < 0.05) between N vs. V, G, F5, F10, and V vs. G, F5, F10, respectively

DL growth leads to a significant increase in angiogenesis in the abdominal and wall regions, providing vascular support to the growing tumor burden in mice [26]. FNC treatment exhibited a dose-dependent inhibition in DL’s ability to induce angiogenesis compared to the Vehicle-treated DL-bearing group (Fig. 4a, b). The F10 treatment group demonstrated 59% less angiogenesis than the vehicle group. The G-group was found to be more effective in controlling DL-induced angiogenesis, exhibiting up to 69% less angiogenesis compared to the vehicle group (p < 0.05).

### FNC upregulated the level of inflammatory cytokine TNF-α

Recent studies have emphasized the role of pro-inflammatory cytokines, such as TNF-α and IL-6, in promoting immune system-mediated tumor clearance [46]. Interestingly, our findings revealed that DL growth can adversely impact the levels of these cytokines. Specifically, TNF-α levels were downregulated from 7.5 (pg/mL) in normal mice to 4.73 (pg/mL) in the Vehicle-treated DL-bearing group, whereas IL-6 levels were slightly upregulated compared to normal mice (Fig. 4c). In contrast, F10 treatment demonstrated a remarkable improvement in TNF-α levels, increasing it to 8.92 (pg/mL) compared to vehicle group. Notably, no significant changes were observed in IL-6 levels in the FNC, and G-group treatments compared to the vehicle group. These results indicate that F10 can significantly modulate the TNF-α in favor of immune-mediated tumor clearance.

### FNC treatment reduces the viability and proliferation of DL cells

**Fig. 5:**
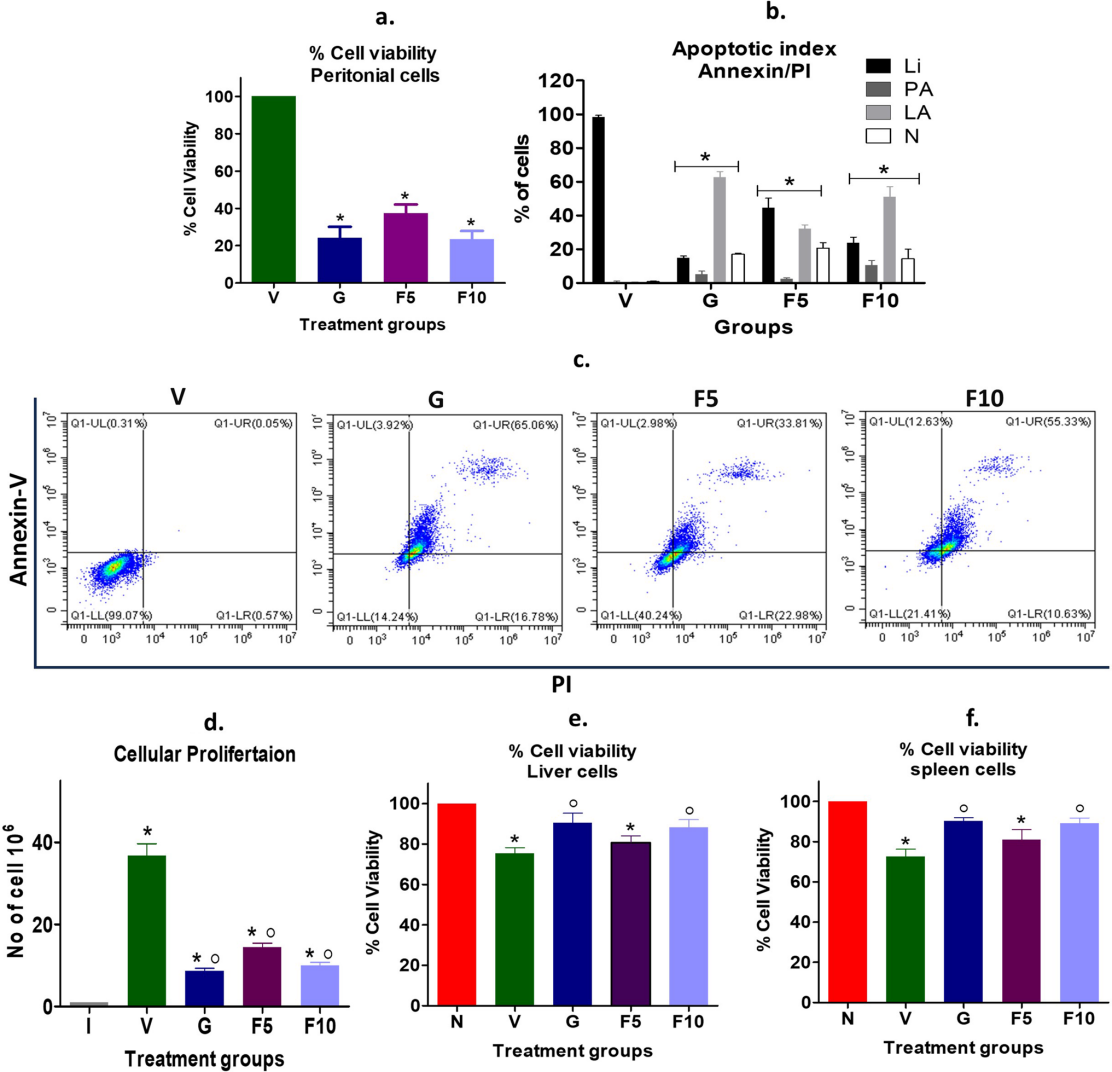
Assessment of cellular viability and proliferation using DCT and Annexin-PI assays. **a**, **e**, **f** The viability of peritoneal cells was determined using the DCT assay. **b**, **c** Annexin/PI staining facilitated the sorting of different peritoneal cell populations based on their viability status, including live (L), pre-apoptotic (PA), late-apoptotic (LA), and necrotic (N) cells. Flow cytometry analysis was performed to characterize these populations. **d** Cellular proliferation was quantified by enumerating the number of live cells in 1 mL of peritoneal fluid, with the initial number of injected cells indicated as “I” in the panel. **e**, **f** Cellular viability of liver and spleen cells was calculated using a DCT assay. The symbolic notation ^○^ and * were used to denote significant differences (p < 0.05) between N vs. V, G, F5, F10, and V vs. G, F5, F10, respectively

FNC treatment has inverted the weight gain in mice and its organs (liver and spleen) due to DL metastasis. Therefore, the viability of peritoneal cells containing DL, and different organs like the liver, and spleen were assessed using the DCT assay. The results showed that the FNC treatment inverting DL mice weight is possibly caused by the reduced viability of DL peritoneal cells. Specifically, the viability reduced to 37%, 24%, and 23% in F5-, F10- and G-groups, respectively, compared to the Vehicle-treated DL-bearing group (Fig. 5a). Furthermore, the annexin-PI study revealed a similarly reduced viability, with a live cell population of 45%, 24% and 15% in F5, F10, and G-groups respectively compared to vehicle group (Fig. 5b, c). The study also showed that most apoptotic cells in the F5- and F10-groups were in the late apoptotic stage, accounting for approximately 50% population. Additionally, the proliferation study demonstrated that, compared to the vehicle group having 36.70 × 10^6^ viable DL cells, the F5, F10, and G-groups had 14.39 × 10^6^, 9.98 × 10^6^, and 8.66 × 10^6^ viable cells, respectively, which confirms the reduced viability of cells (Fig. 5d). DL growth known to reduce the viability of the liver and spleen due to metastasis foci, and treatment with F10 and G-groups exhibited significantly enhanced viability than vehicle group (Fig. 5e, f). The findings suggest that FNC treatment can effectively inhibit DL cell viability and proliferation, which may contribute to the reduced weight of organs affected by metastasis.

#### FNC reduced DL proliferation through apoptosis

**Fig. 6:**
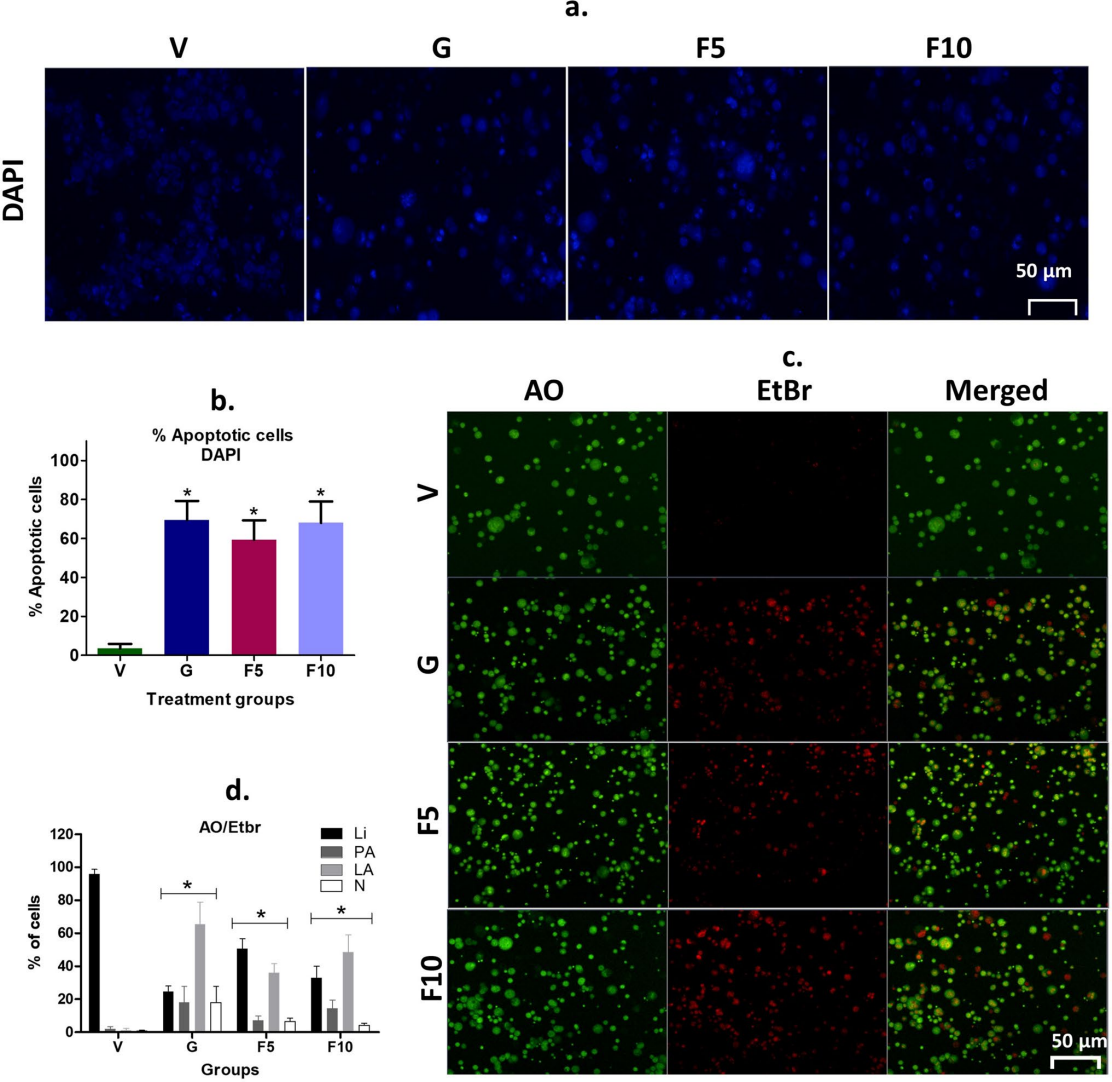
DAPI and AO/EtBr staining. **a** DAPI staining images were obtained using a fluorescence microscope. **b** Calculation of apoptotic cell indexes by quantifying 300 cells. **c** Utilization of AO/EtBr staining to classify cells into different stages of apoptosis, including live (L), pre-apoptotic (PA), late-apoptotic (LA), and necrotic (N) cells, visualized under a fluorescence microscope. **d** Computation of indexes based on the counting of 300 cells from panel **c**. A scale bar of 50 μm was utilized to represent the image size. The symbolic notation * was used to indicate significant differences (p < 0.05) between V vs. G, F5, and F10, respectively

**Fig. 7:**
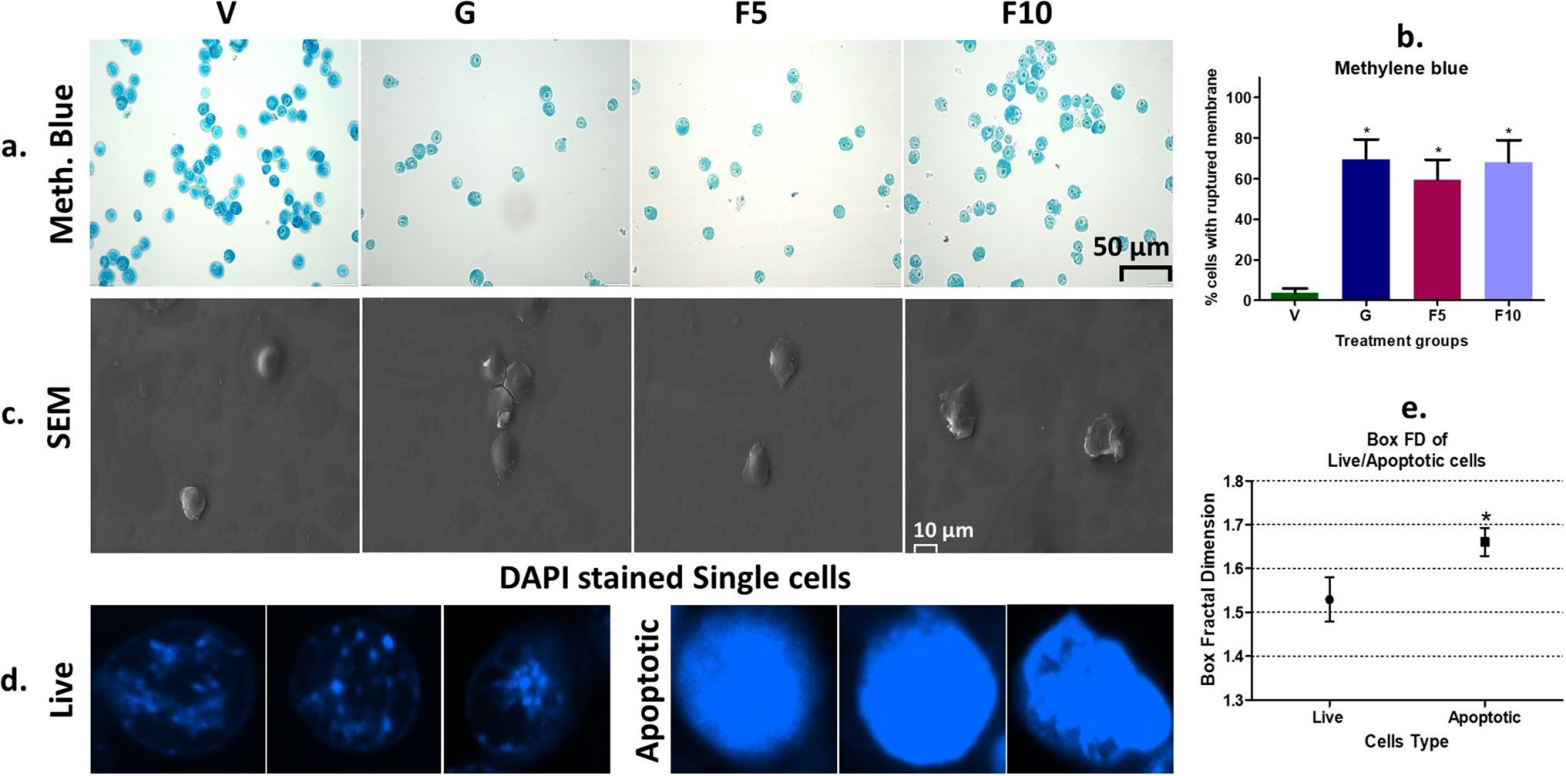
Methylene blue staining, SEM, and Box-FD counting. **a** Methylene blue staining photographs were captured on a fluorescence microscope using the bright field mode. The scale bar of 50 μm was employed for the analysis. **b** Calculation of indexes by counting 300 cells. **c** SEM imaging of the peritoneal cells from different groups, with a scale bar of 10 μm used for the analysis (magnification ×2000). **d**, **e** Additionally, Box-FD values for live and apoptotic cells were calculated using Image J software. Symbolic notation * indicates significant differences (p < 0.05) between V vs. G, F5, F10, and live and apoptotic cells, respectively

Fluorescent dyes-based tests were performed to investigate whether FNC treatment reduces DL growth and proliferation through apoptosis. DAPI staining revealed a significant population of cells with DNA fragmentation and chromatin condensation in the FNC groups, compared to the Vehicle-treated DL-bearing group (Fig. 6a, b). Additionally, AO/EtBr staining indicated a higher number of cells that lost membrane integrity, allowing EtBr staining, compared to the vehicle group (Fig. 6c, d). The number of late apoptotic cells in AO/EtBr staining was higher in the F10-group than in the F5-groups, which supports Annexin V/PI findings. As a rule, fluorescent tests should be further confirmed by non-fluorescent tests. Therefore, Methylene blue staining was used, which showed that cells with apoptotic characteristics increased with FNC concentration (Fig. 7a, b). SEM analysis revealed that DL cells had a comparatively rounded shape, while apoptotic cells showed a distorted shape, and disrupted membrane (Fig. 7c). Furthermore, analysis of live and apoptotic cells using Box-FD indicated significant quantitative differences, with live cells having a Box-FD of 1.52 and dead cells having a Box-FD of 1.66 (p < 0.05) (Fig. 7d, e). Box-FD is a useful tool for identifying different cell types based on different textures, where increased Box-FD indicates that cells have lost their fine and smooth surface texture and moved to a grainer and coarser irregular surface texture due to apoptotic blebs and loss of membrane structure [4].

### FNC diminished the level of mitochondrial membrane potential (MMP) in the DL cells

**Fig. 8:**
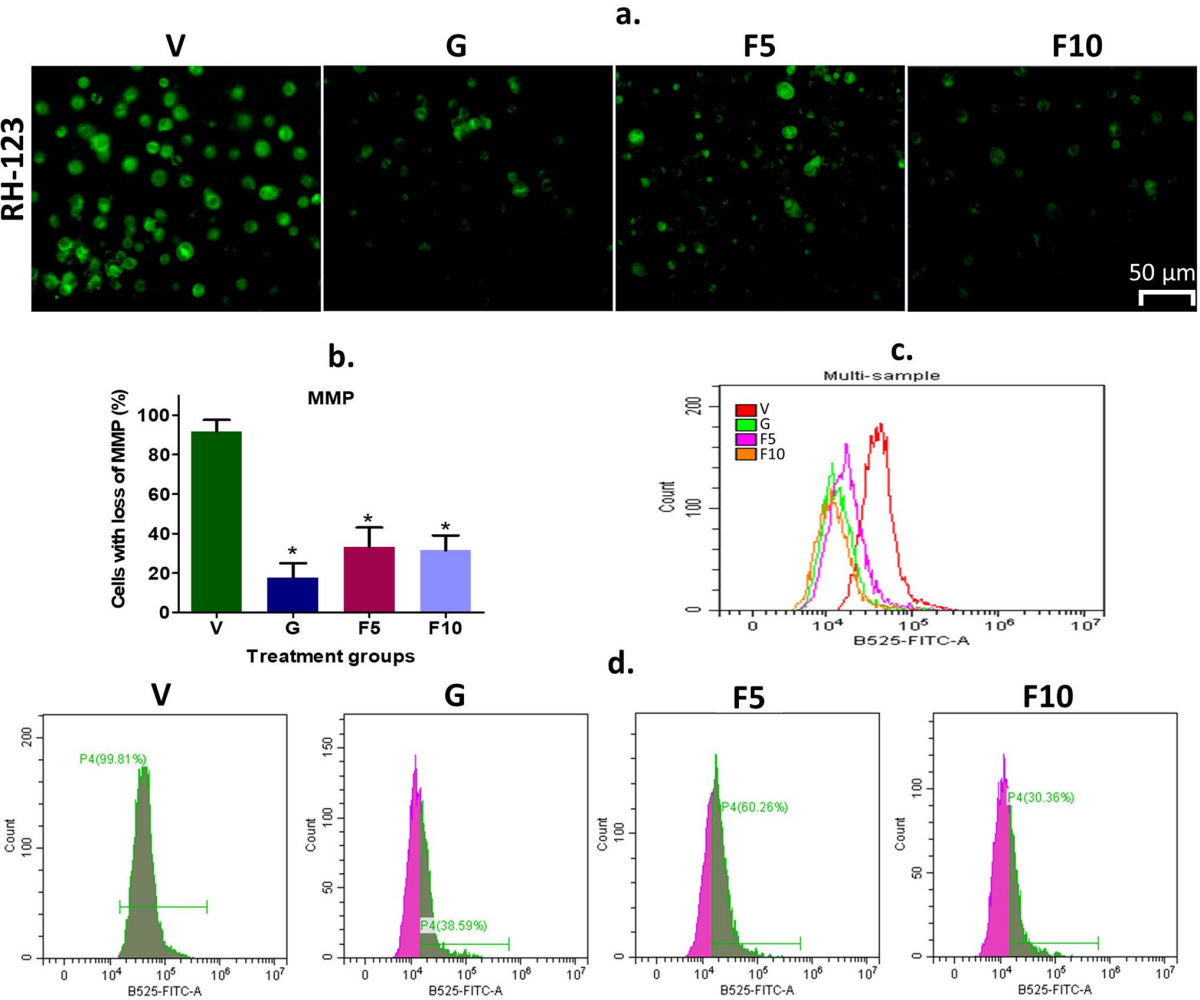
Visualization and quantification of MMP in cells. **a** Representative Rh-123 fluorescence microscopy images depicting MMP levels. The scale bar represents 50 μm. **b** Quantification of MMP indexes based on the analysis of 300 cells. **c** Comparative assessment of overall MMP changes measured by flow cytometry in each experimental group. **d** Individual sample analysis showing changes in MMP levels measured by flow cytometry. Symbolic notation * indicates significant differences (p < 0.05) between V vs. G, F5, and F10

The reduction of MMP is a hallmark of apoptosis [25]. To investigate whether FNC treatment induces apoptosis through MMP reduction, Rh-123 dye was used. Fluorescence microscopy qualitative analysis revealed that an increasing concentration of FNC resulted in a greater number of cells with lower levels of MMP compared to the Vehicle-treated DL-bearing group (Fig. 8a, b). Flow cytometry-based quantitative analysis demonstrated that a significant reduction of MMP observed in treatment groups with 39%, 60%, and 30% MMP levels was observed respectively in G, F5, and F10-groups, compared to the vehicle group (p < 0.05) (Fig. 8c, d). This result showed that FNC treatment induces apoptosis in DL cells through MMP reduction.

### FNC induces apoptosis through the induction of reactive oxygen species (ROS) in DL cells

**Fig. 9:**
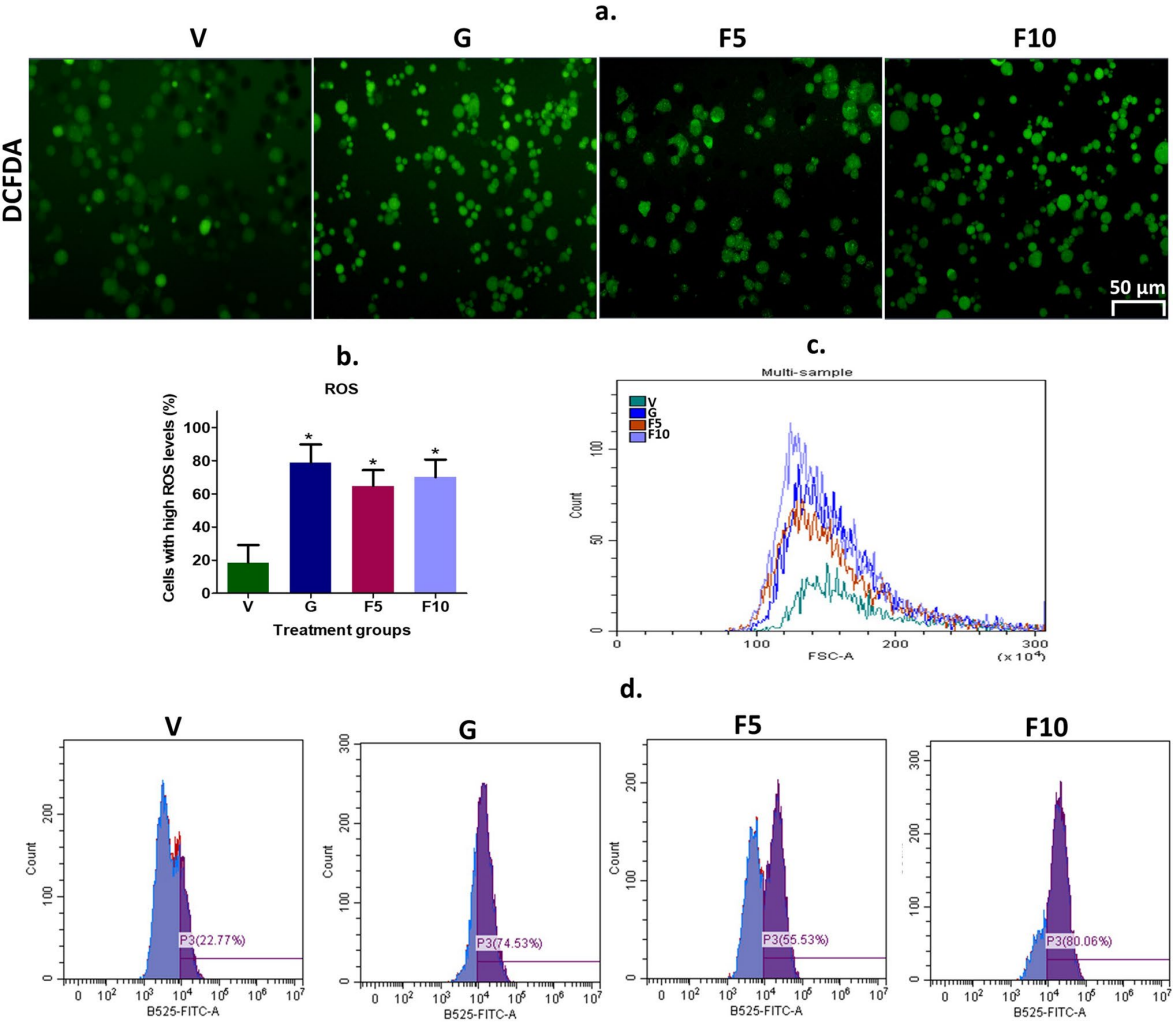
Visualization and quantification of ROS in cells. **a** Representative fluorescence microscopy images of ROS levels using DCFDA staining. The scale bar represents 50 μm. **b** Quantification of ROS indexes based on the analysis of 300 cells. **c** Comparative assessment of overall ROS changes measured by flow cytometry in each experimental group. **d** Individual sample analysis demonstrates alterations in ROS levels measured by flow cytometry. Symbolic notation * indicates significant differences (p < 0.05) between V vs. G, F5, and F10

The reduced viability of DL cells observed after treatment with FNA may be due to the potent activation of ROS [52]. To investigate this, the ROS assay was performed using DCFDA dye. Fluorescence microscopy revealed a significant increase in ROS levels in treatment groups compared to the Vehicle-treated DL-bearing group (Fig. 9a, b). Flow cytometer analysis showed 74%, 55%, and 84% of ROS induction in the G, F5, and F10-groups, respectively, compared to the vehicle group (p < 0.05) (Fig. 9c, d). Therefore, ROS may be one of the key factors behind the observed reduced viability of DL cells after FNC treatment.

### FNC augmented the mean survival time and increased the lifespan of DL mice

**Fig. 10:**
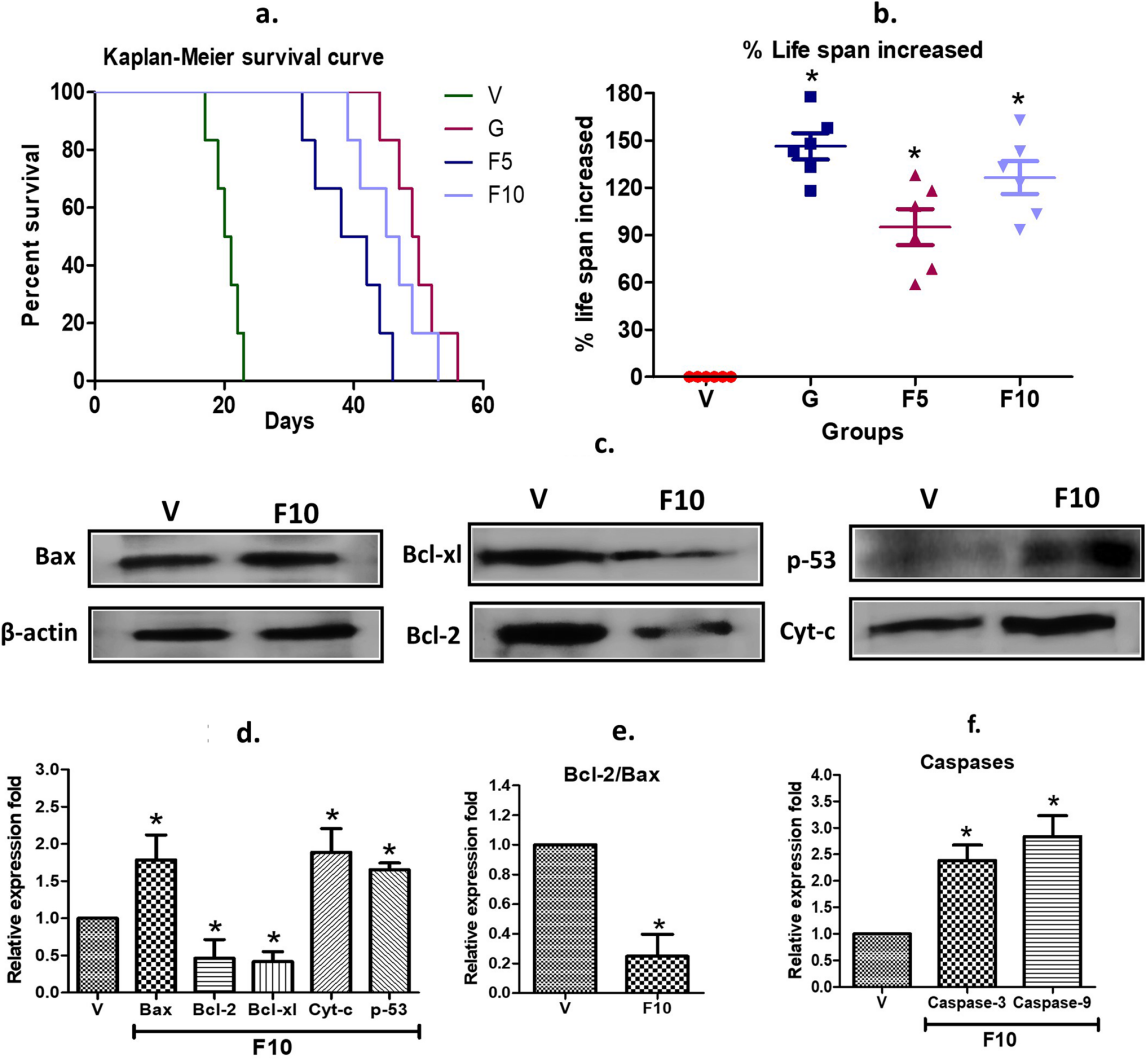
Survival study and analysis of apoptotic factors. **a** Kaplan–Meier survival curve illustrating the survival data of mice. **b** Calculation of the percentage increase in lifespan using the formula (T − C/C) × 100, where T and C represent the survival time in days for treatment and control (DL) mice, respectively. **c** Represents western blotting analysis of the effects of FNC on apoptosis-related factors in DL cells. **d** Fold change in protein expression of various genes associated with apoptosis in mitochondria, including Bax, Bcl-2, Bcl-xl, Cyt-c, and p-53. **e** The ratio of Bcl-2/Bax protein expression. **f** Results obtained from kit-based analysis of caspase-3 and -9. Statistical significance (*p < 0.05) is indicated by symbolic notation: * for significant differences between V and G, F5, and F10 in terms of increased lifespan, and between V and F10

The inhibitory effect of FNC on the growth and proliferation of DL cells was found to be promising in vivo, as it led to a significant increase in the mean survival time to 46 days in F10 treatment mice from 20.5 days in Vehicle-treated DL-bearing group (Fig. 10a). The survival time of the F10-group was almost comparable to that of the G-group, which exhibited a mean survival time of 49.5 days. Remarkably, both F10 and G-group treatments led to a significant extension of the lifespan of DL mice. Specifically, both treatments resulted in a 124–141% increase in survival compared to the vehicle group, whose lifespan was considered as 0% for comparison (Fig. 10b). These results indicate the potential of FNC as a therapeutic agent for the treatment of DL cancer. Further investigations, therefore, were warranted to explore its translational significance in the clinical setting.

### FNC induces mitochondrial-mediated apoptosis in DL cells

The reduction in MMP observed in fluorescents tests (Sect. 3.6, Fig. 8) suggested that FNC treatment might induce mitochondrial-mediated apoptosis in DL cells. Therefore, the expression of various apoptosis-related proteins, including cytochrome-c, Bax, p-53, and caspases 3, and 9, were examined. The results indicated a significant increase in the expression of cytochrome-c, Bax, p-53, and caspases 3, and 9 in the FNC-treated group compared to the Vehicle-treated DL-bearing group (Fig. 10c, d). In contrast, FNC treatment (F10) decreased the expression of Bcl2 and Bcl-xl protein, compared to the vehicle group. The decreased Bcl2 level and increased Bax levels lead to a decreased Bcl-2/Bax ratio (Fig. 10e), explaining the release of cytochrome-c and increased caspase levels inducing cells’ death to FNC treatment. The upregulation of cytochrome-c and caspases 3 and 9 (Fig. 10f), together with the downregulation of Bcl2 and Bcl-xl, suggested that FNC treatment might activate the intrinsic apoptotic pathway in DL cells. The intrinsic pathway is initiated by the release of cytochrome-c from the mitochondria, which forms a complex with Apaf-1 and procaspase-9, leading to the activation of caspase-9. Activated caspase-9 then activates caspase-3, which ultimately leads to apoptotic cell death. Moreover, the upregulation of p-53 in FNC-treated DL cells suggests that FNC might induce apoptosis through a p-53-mediated pathway. p-53 is known to play a crucial role in the regulation of apoptosis by inducing the expression of pro-apoptotic genes such as Bax and downregulating anti-apoptotic genes such as Bcl2.

## Discussion

FNAs mimic natural purine and pyrimidine compounds and disrupt cellular pathways, making them potent anti-cancer agents [2, 20]. They have shown promise in treating various cancers, including lymphoma, leukemia, and solid tumors [32]. In this study, we investigated the anti-cancer activities of a specific FNA called FNC in the DL model system, which is a relevant model for human T-cell lymphoma (Fig. 1) [18, 23].

Our results revealed that the F10-group effectively prevented alteration in various behavioral parameters, including food and water intake, which were previously reported to be seriously altered due to DL progression (Table 2) [39]. Moreover, DL progression has been associated with a marked increase in body weight due to the high protein requirements of the tumor cells [3, 35, 44]. We observed a less body weight gain in the FNC-treated group (Fig. 2), indicating a potential inhibitory effect of FNC on DL-induced metabolic alterations.

DL metastasis to the liver can lead to the formation of metastatic foci, altering liver structure and enzyme configuration [40]. Increased levels of liver markers enzymes SGOT and SGPT, as well as bilirubin, in DL hosts (Table 4) suggest liver damage or dysfunction caused by DL proliferation and metastasis [19]. Interestingly, FNC treatment prevented DL-induced alterations in liver enzymes and bilirubin levels, effectively protecting the liver from DL-induced injury. Moreover, FNC treatment prevented the development of metastatic foci in the liver (Fig. 3), suggesting its potential to mitigate structural damage caused by DL metastasis. The maximum absorption of FNC in the spleen and liver further supports its potential implications for the treatment of cancer-induced liver injury [42].

DL significantly alters various blood parameters to favor its growth [33, 36]. Our recent studies have revealed that DL growth results in changes to the levels of WBC, RBC, hemoglobin (Hb), platelets, neutrophils, lymphocytes, and mean corpuscular hemoglobin concentration (MCHC) [22, 40]. Remarkably, FNC treatment counteracted these changes caused by DL (Table 3). Additionally, DL growth is associated with neo-vascularization, a critical process that enables cancer cells to proliferate and metastasize [17]. We found that FNC treatment significantly inhibited DL-induced vascularization (Fig. 4), suggesting its potential to impede tumor growth and metastasis.

Apoptosis, a process of programmed cell death, plays a vital role in regulating the life cycle of cells. It is a complex and highly orchestrated process characterized by specific morphological and biochemical changes [29]. FNC treatment was found to induce apoptosis in DL cells to reduce their growth and proliferation (Figs. 5, 6 and 7). Apoptosis can be initiated by various cellular stresses, including oxidative stress, mitochondrial depolarization, or DNA damage [43]. Cancer cells have a high metabolic demand due to the hyperpolarization of the mitochondrial membrane, which is required to meet their excessive energy needs. This results in high MMP in cancer cells, making it a promising target for cancer therapy [10]. Interestingly, FNC has been found to significantly reduce the MMP level in DL cells, thereby triggering apoptosis by lowering MMP (Fig. 8). Moreover, tumor growth is highly dependent on aerobic glycolysis, known as the Warburg effect, which is associated with increased oxidative stress or ROS levels. High ROS levels can damage cellular components, including DNA and metabolic proteins, leading to cell death through apoptosis [31, 37]. Most cancer chemotherapeutics induce cell death by increasing ROS levels above a certain threshold [52]. In this context, it is noteworthy that FNC treatment caused a dose-dependent increase in ROS levels in DL cells (Fig. 9), indicating that ROS induction might be one of the key factors underlying the observed apoptosis by FNC. These findings provide an important insight into the molecular mechanisms of FNC-induced apoptosis in cancer cells.

Apoptosis is a highly regulated, programmed cell death process that is essential for normal development and tissue homeostasis. It is a complex process that involves a variety of molecular signalling pathways and effector molecules. Mitochondria play a crucial role in the initiation and execution of apoptosis, and mitochondrial dysfunction is a key factor in various diseases, including cancer. Mitochondrial-mediated apoptosis is a well-known pathway that involves the release of cytochrome-c from the mitochondria into the cytosol, which triggers the activation of caspases and the initiation of apoptotic signalling [50]. The Bcl-2 family of proteins plays a crucial role in regulating mitochondrial-mediated apoptosis by regulating the permeability of the mitochondrial outer membrane, which alters the cell’s MMP level. Pro-apoptotic members of the Bcl-2 family, such as Bax, Bak, and Bid, promote mitochondrial permeabilization, while anti-apoptotic members, such as Bcl-2 and Bcl-xL, inhibit it [16]. In the present study, FNC was found to induce mitochondrial-mediated apoptosis in DL cells by reducing MMP, which leads to the release of cytochrome-c and the activation of caspases. The western blotting analysis showed an increased expression of the pro-apoptotic protein Bax and a decreased expression of anti-apoptotic protein Bcl2 in the F10-group, indicating the activation of the mitochondrial-mediated apoptosis pathway (Fig. 10). The increased levels of cytochrome-c and Caspases 3, and 9 further confirmed the activation of mitochondrial-mediated apoptosis. These findings are consistent with recent studies in NSCLC H460 cell line and Lewis’s mouse model [13]d cell’s NHL cell lines RL, Granta-519, SUDHL-6 [47], suggesting the potential of FNC as an anti-cancer agent by inducing apoptosis through the mitochondrial-mediated pathway.

FNC’s ability to suppress DL cell proliferation and induce apoptosis suggests its potential as a promising alternative or complementary treatment for cancer or NHLs. The observed reduction in DL cell growth and proliferation in response to FNC treatment correlates with the increase in apoptosis induced by the treatment, therefore it is plausible to assume that FNC treatment might increase the life span of DL mice. Interestingly, FNC treatment showed a significant increase in the survival rate of mice bearing DL cells. The mean survival rate of mice increased from 20.5 to 46 days, indicating an overall life span extension of 124%, a remarkable result as compared to the vehicle group (Fig. 10). In comparison, the standard chemotherapy drug gemcitabine increased the survival rate of mice by 49.5 days, which is comparable to the effects of FNC treatment. These findings are consistent with the results reported in previous studies on FNC’s efficacy against other cancer types, such as NSCLC and B-cell’s NHL cell lines [13]; Wang et al. [47]. Overall, these results indicate that FNC has the potential to be a promising candidate for future DL cancer treatments.

## Conclusion

**Fig. 11:**
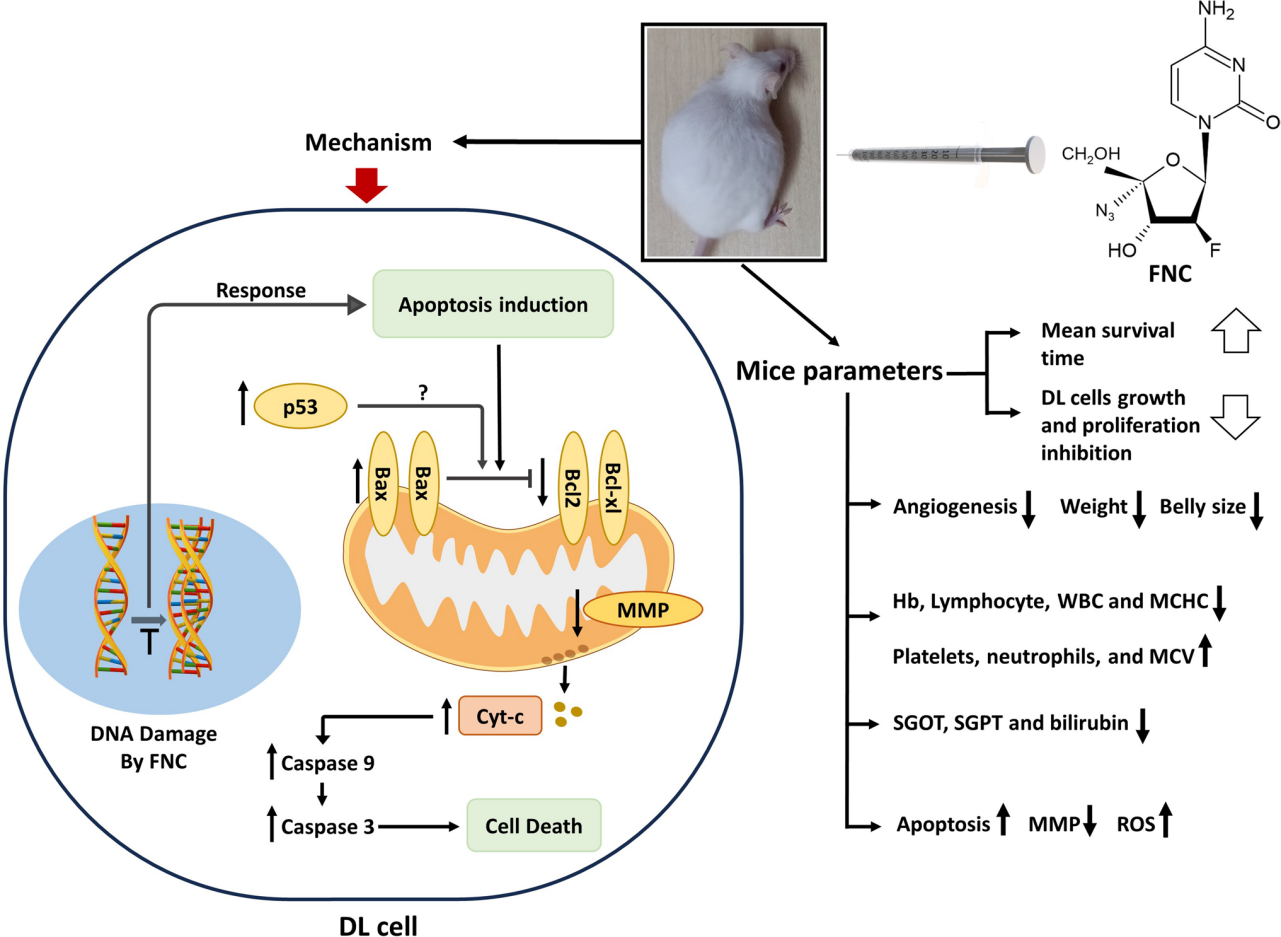
Schematic representation of the in vivo anti-tumor activity of FNC against DL. FNC increases the mean survival time of mice by inhibiting DL growth through decreasing/increasing various DL-induced factors. The mechanism of action involves apoptosis induction via a mitochondrial-mediated pathway involving increased expression of apoptotic factors Bax, the release of cytochrome-c and caspase 3, 9, and decreased expression of anti-apoptotic factors Bcl2 and Bcl-xl. The possible cause of apoptosis is ROS generation followed by decreased MMP

In this study, the anti-cancer activity of the FNA, FNC was investigated in a mouse model of T-cell lymphoma called DL. This study demonstrates that FNC exhibits anti-cancer activities in a mouse model of T-cell lymphoma. The findings suggest that FNC may have a multifaceted impact on DL progression, including the inhibition of neo-vascularization, regulation of cytokine levels, ROS production, induction of mitochondrial-mediated apoptosis through MMP disruption, and protection of liver function (Fig. 11). Furthermore, FNC treatment resulted in significant improvement in behavioral and haematological parameters and extended the survival of DL-bearing mice. These results indicate that FNC may hold promise as a potential therapeutic agent for the treatment of T-cell lymphoma and warrant further investigation in clinical trials.

## Data Availability

This published article encompasses all the data gathered throughout this experimental study, reflecting a comprehensive collection across multiple sets. This published article includes all of the data collected during this experimental study.
