# Correction: Mitochondrial-mediated apoptosis as a therapeutic target for FNC (2′-deoxy-2′-b-fluoro-4′-azidocytidine)-induced inhibition of Dalton’s lymphoma growth and proliferation

**DOI:** 10.1007/s12672-026-05021-0

**Published:** 2026-05-06

**Authors:** Naveen Kumar, Sanjeev Kumar, Alok Shukla, Sanjay Kumar, Rishi Kant Singh, Ilya Ulasov, Sandeep Kumar, Anand Kumar Patel, Lokesh Yadav, Ruchi Tiwari, Rachana, Shivashish Priyadarshi Mohanta, Kaushalendra, Vikram Delu, Arbind Acharya

**Affiliations:** 1 Department of Zoology, Institute of Science, Banaras Hindu University, Varanasi, Uttar Pradesh 221005 India; 2 Department of Zoology, Lucknow University, Lucknow, Uttar Pradesh 226007 India; 3 Group of Experimental Biotherapy and Diagnostic, Department of Advanced Materials, Institute for Regenerative Medicine, Sechenov First Moscow State Medical University, Moscow, 119991 Russia; 4 World-Class Research Center, Digital Biodesign and Personalized Healthcare, Sechenov First Moscow State Medical University, Moscow, 119991 Russia; 5 Department of Zoology, Pachhunga University College Campus, Mizoram University, Aizawl, Mizoram 796001 India; 6 Haryana State Biodiversity Board, Panchkula, Haryana 134109 India

**Correction: Discover Oncology (2024) 15:16** 10.1007/s12672-023-00829-6

Following publication of our article [1] in *Discover Oncology* (January 2024), it has come to our attention that two figure panels contained inadvertent assembly and labeling errors during final preparation of the figures.

**Correction 1—**Fig. 3a


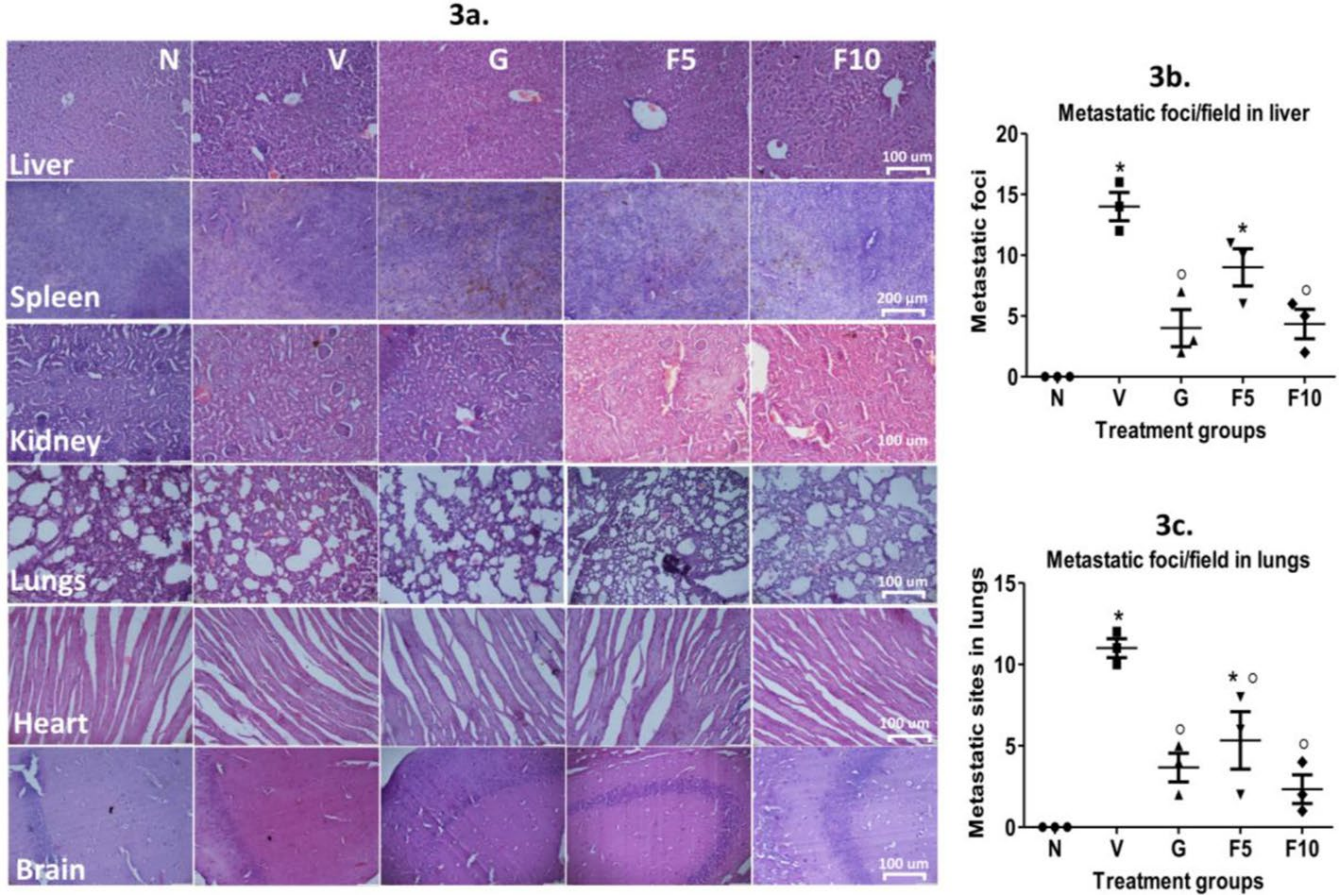


In Fig. 3a, the panel labeled as **F5** inadvertently contained a **20×micrograph of the G group**, while all other panels in this figure were captured at **40×magnification**.

This misplacement occurred during the figure-assembly stage when the 20×image of the G group—taken only for internal comparison—was mistakenly inserted in the position intended for the F5 group.

We have now corrected this by replacing the misplaced 20×image with the **verified 40×micrograph corresponding to the F5 treatment group**. The corrected Fig. 3a now uniformly represents all treatment groups at 40×magnification.

**Correction 2—**Fig. 6c


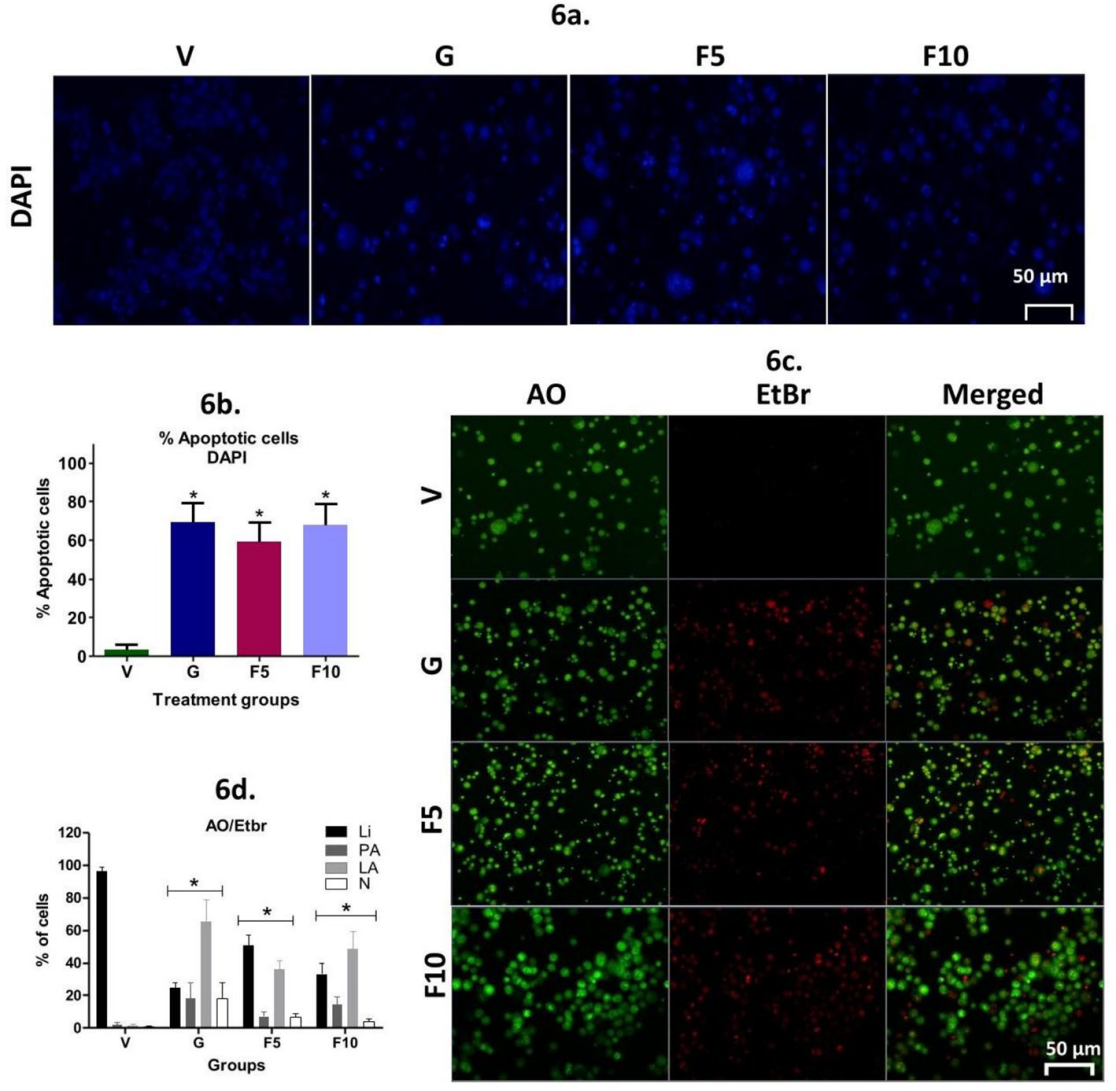


In Fig. 6c, the micrographs for F5 and F10 treatment groups were also affected by a layout oversight. During figure preparation, a representative image of the F5 group was temporarily placed beside other groups for comparison and selection of the best field, but this preliminary image was inadvertently retained and labeled as F10 in the final version.

The correct high-resolution micrograph corresponding to the F10 treatment group has now replaced this placeholder image. The updated Fig. 6c accurately displays distinct fields for each treatment group as intended.

These issues arose solely from **figure-placement and labeling errors** during assembly and **do not affect any data, analyses, or conclusions** presented in the article.

The corrected figures are provided for reference.

We sincerely thank the readers who brought these matters to our attention and the editorial team for the opportunity to correct the record.

**Data availability**

The datasets and original image files supporting the corrections presented in this correction are available from the corresponding author upon reasonable request.

On behalf of authors,

Prof. Arbind Acharya (Corresponding Author)

Department of Zoology, Institute of Science, Banaras Hindu University, Varanasi, India

Email: acharya@bhu.ac.in
